# Evaluating the Treatment of Diabetic Macular Edema with Aflibercept Based on a Regional Network of Ophthalmologic Care Givers

**DOI:** 10.1155/2023/3165965

**Published:** 2023-01-10

**Authors:** Haidar Khalil, Siegfried Mariacher, Rupert Strauss, Dominika Podkowinski, Klemens Waser, Matthias Bolz

**Affiliations:** ^1^Department of Ophthalmology and Optometry, Kepler University Hospital, Linz 4020, Austria; ^2^Department of Ophthalmology, Medical University Graz, Graz 8036, Austria; ^3^Vienna Institute for Research in Ocular Surgery (VIROS), Hanusch Hospital, Vienna 1140, Austria

## Abstract

**Purpose:**

In Austria, anti-VEGF therapies are reimbursed only in clinical settings. This study aimed to describe the outcome of a treat and extend regimen (TER) with aflibercept for diabetic macular edema (DME) in a network of practitioners.

**Methods:**

In a prospective study over 36 months, patients with DME were treated with a loading dose of aflibercept and further on with adjusted treatment intervals based on optical coherence tomography (OCT) findings. All patients were monitored in an outpatient setting by regional ophthalmologists, and the treatment was administered in the clinic. Main outcome parameters were best-corrected visual acuity (BCVA) from baseline to the last regular visit. Number of visits at the practitioner's office as well as the number of injections were secondary outcome parameters.

**Results:**

Thirty-three patients completed the study at their final visit. BCVA improved significantly by 5.8 letters between baseline and the final visit from 70.4 letters at baseline (*p*=0.004). Patients visited the practitioner's office 12.8 times in the observation period of 36 months. 3.7, 5.1, and 3.9 visits were performed, respectively, in the first, second, and third years, and 25.5 ± 7.9 injections were performed. The mean interval of injections over the observation period was 6.2 ± 2.2 in weeks.

**Conclusion:**

The treat and extend regimen was valuable for treating patients with DME in this specific setting. The functional results of this study were comparable to those of other real-world evaluations. Adherence to the same treating institution seems to be important to avoid differences in therapeutic decision making and may also increase patient's compliance.

## 1. Introduction

Diabetic macular edema (DME) is one of the major causes of vision loss in developed countries and the leading cause of vision loss in patients with diabetes type 1 and type 2, affecting 1 in 15 people with diabetes [1]. Anti-VEGF (anti-vascular endothelial growth factor) agents are the gold standard in the treatment of DME. Compared to laser monotherapy, anti-VEGF intravitreal therapy showed superior BCVA (best-corrected visual acuity) achievements in multiple studies [2, 3]. Aflibercept, bevacizumab, and ranibizumab showed improvement of BCVA depending on initial vision loss, whereas aflibercept was more effective than the other two agents in patients with severe vision loss [4]. The two main concepts to administer anti-VEGF therapy in clinical practice are either PRN (pro re nata) or TER (treat and extend regimen). In PRN, treatment is only conducted in case of recurring macular edema while visits are performed on a regular basis, usually every four weeks, whereas in TER, the interval of the visits will be extended or reduced individually for each patient depending on the activity of the disease. The advantage of TER is reducing the number of visits and avoiding over- and under-treatment.

In Austria, anti-VEGF therapy is only reimbursed in a clinical setting and not in out-clinic offices, leading to a significant organizational burden for ophthalmological departments. Even more, the overall requirement of intravitreal injection is exponentially growing throughout the last decade. For this reason, a network of caretakers of a university clinic and of ophthalmologic practitioners were established to avoid redundant examinations and define a disease management protocol for the anti-VEGF treatment of patients with DME. Based on this protocol, patients were followed by practitioners indicating the individual treatment interval, and all the treatments were performed at the university clinic.

This study aimed to evaluate a TER with aflibercept in this network of intra and extramural caregivers. TER was chosen to lower disease burden of the patients and for evaluation of patients' adherence to this specific regimen. Morphologic and functional results and the efficacy of the treatment protocol were analyzed in detail.

## 2. Materials and Methods

### 2.1. Study Design

The study was a monocentered, prospective, open-labeled phase 4 trial investigating a standard TER with aflibercept (Eylea, Bayer Pharma AG, 2 mg/0.05 ml per injection). The study followed the tenets of the Helsinki agreement and was approved by the local Ethics Committee.

### 2.2. Inclusion and Exclusion Criteria

Patients with DME were recruited from the outpatient clinic of the Dpt. of Ophthalmology of the Kepler University Clinic, Linz, Austria as participants of the study. They were either referred by external ophthalmologists or by self-referral. Main inclusion criteria were as follows (1) patients with diabetes type 1 or 2; (2) the presence of intra or subretinal fluid within the central 6 mm and central subfield macular thickness ≥300 *μ*m, as determined by the optical coherence tomography (OCT; Spectralis OCT®, Heidelberg Engineering, Dossenheim, Germany); (3) best-corrected visual acuity (BCVA) >30 letters based on the ETDRS charts. If both eyes were affected, the eye with worse BCVA was included in the study. Both proliferative and nonproliferative stages of diabetic retinopathy were included. The need for or previous laser coagulation was not exclusion criteria. Before being admitted to this study, the subject must have consented to participate after the nature, scope, and possible consequences of the clinical study have been explained in an understandable way.

Key exclusion criteria were any prior treatment of DME within 3 months before inclusion, other reasons for macular edema or visual impairment except for ametropia, inability to communicate in German or English, and active intra or periocular infection in the study of eye or hypersensitivity to aflibercept.

### 2.3. Treatment and Assessment

**Figure 1:**
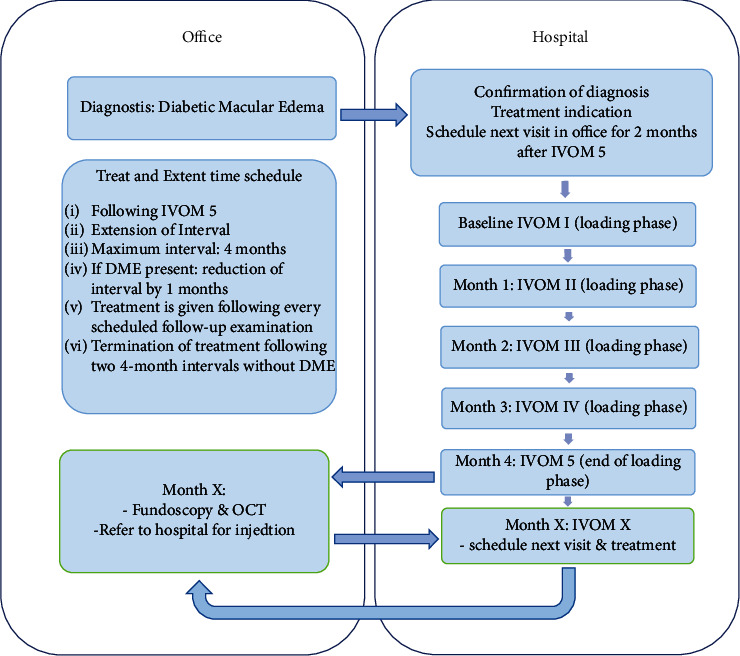
After a patient was included in the study, 5 monthly injections were admitted as loading dose. 2 months after the fifth injection, the patient went to the practitioner's office for OCT scan and funduscopic examination. If a macular edema is present, the patient was referred to our clinic for reevaluation and injection if indicated.

Treatment with aflibercept was initiated with a loading dose consisting of 5 monthly injections with aflibercept at the department of ophthalmology, Kepler University Clinic, Linz, Austria. Following this loading dose, treatment intervals were extended or shortened continuously according to TER based on individual OCT scans. If there was no sign or no change of intraretinal fluid of more than 10% in two follow-up visits in the central 6 millimeters as assessed by the EDTRS grid in macular OCT reports, the interval was extended in steps of 4 weeks to a maximum of 16 weeks. If a follow-up investigation revealed the presence of increasing or constant DME involving the central 6 millimeters, the interval was reduced by 4 weeks. Intervals were either shortened or extended in steps of 4 weeks (minimum 4 weeks and maximum 16 weeks). If the maximum interval of 16 weeks was achieved twice, the treatment was ceased (Figure 1). Following the loading dose, depending on the presence of DME, the practitioner decided whether the treatment interval should be shortened or extended. To evaluate the individual treatment decision in the network of caregivers in offices and in the clinical centers, each follow-up visit was performed twice, at the outpatient practitioner's office and at the study center of the Kepler University clinic. All the treatments were performed at the university clinic.

At each follow-up, a full-ophthalmologic examination was performed with fundus examination, OCT scan, and BCVA. Fluorescein angiography (FA) was performed at the baseline, after 6, 12, and 24 months using 10% injectable solution fluorescein sodium (SERB, Paris, France). Standardized OCT measurements were performed using the Spectralis HRA + OCT system (Heidelberg Engineering, Heidelberg, Germany).

### 2.4. Outcome Measures

The change in the BCVA from the baseline to the final visit was the primary end point. The secondary outcome measures were CRT injection interval, number of visits at the clinic, and the practitioner's office.

### 2.5. Statistical Analysis

Wilcoxon signed-rank or Mann-Whitney U nonparametric tests were used to compare the measured values. Categorical variables were analyzed using the *χ*^2^ test or Fisher's exact test. The statistical significance was defined as *p* < 0.05. IBM SPSS Statistics for Windows (v23.0, IBM Corp., Armonk, NY, USA) was used to perform statistical analyses.

## 3. Results

Forty-one patients were enrolled, of which 33 finished the study according to the study criteria. Twenty-five patients completed the study on the final visit at month 36 (Table 1). Eight patients had their final visit before month 36, because the injection interval was extended twice to 16 weeks. Eight patients could not complete the study (death *n* = 1, termination of patient *n* = 3, cardiorespiratory diseases that lead to study dropout *n* = 4).

**Figure 2:**
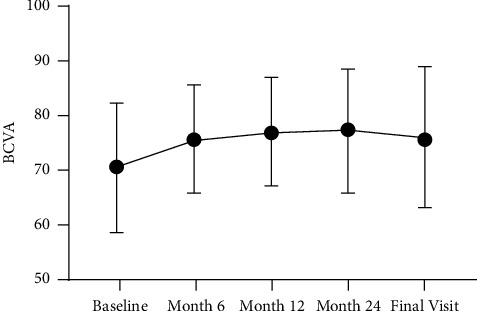
BCVA over the observation period of all patients who completed the study.

**Figure 3:**
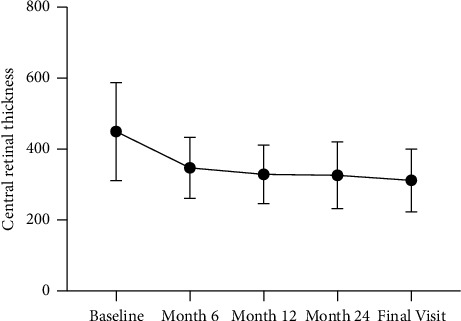
CRT over the observation period of all patients who completed the study.

BCVA improved significantly from 70.4 ± 11.9 letters at the baseline to 76.2 ± 13.0 letters at the final visit (+5.8 ± 13.4 letters, 95% confidence interval 1.0 to 10.5 letters, *p*=0.019). Significant BCVA changes were observed until month 6 (+6.3 letters ± 10.0, *p* < 0.001). Thereafter, no additional significant changes could be found (Figure 2). CRT reduced significantly from 465.8 ± 145.9 *μ*m at the baseline to 311.8 ± 88.6 *μ*m at the final visit (−154.1 ± 151.3 *μ*m, *p* < 0.001) (Figure 3).

**Figure 4:**
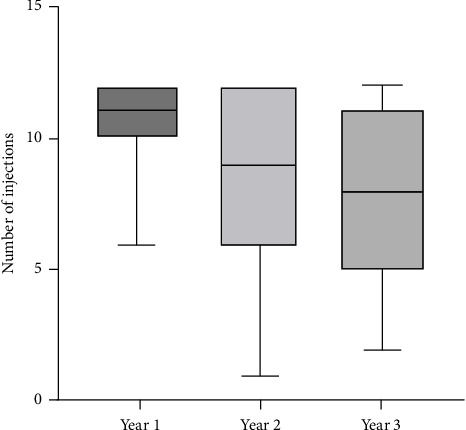
Number of injections admitted to patients who completed the study in the first, second and, third years.

Over the follow-up of 36 months, the mean number of injections was 25.5 ± 7.9. In the first, second, and third years, 10.8 ± 1.5, 8.6 ± 3.3, and 7.9 ± 3.3 injections were performed, respectively. Thus, the number of injections decreased significantly from the first year to the third year by 2.9 injections (±3.3, 95% confidence interval 1.6 to 4.2, *p* < 0.001) (Figure 4).

The mean interval between the follow-up visits was 6.2 ± 2.2 weeks over the observation period. In the first year, the mean interval (weeks) between injections following the loading dose was 5.0 ± 1.5 (median 4, range 4 to 8), 7.2 ± 3.9 (median 5.3, range 4 to 16) in the second year and 8.1 ± 4.4 (median 6.0, range 4 to 16) in the third year. The interval increased significantly from the first year to the third year by 3.2 weeks (±4.1, 95% confidence interval 1.7 to 4.8, *p* < 0.001).

Patients visited the practitioner's office 12.8 ± 10.6 times in the observation period. In the first year, 3.7 ± visits were performed, 5.1 ± 4.5 in the second year, and 3.9 ± 4.3 in the third year.

Seventeen patients visited the practitioner's office more than 6 times a year in the observation period. Those patients had altogether 431 visits to their ophthalmologist. The decision of the external ophthalmologist was congruent with the decision of the clinic in 86% to admit an intravitreal injection (*n* = 372). In 14% (*n* = 59), the decision differed. In 32% (*n* = 19) of the incongruent decisions, the practitioner indicated a therapy, as opposed to the clinic. In 68% (*n* = 40) of the incongruent decisions, the clinic indicated an injection, as opposed to the practitioner. However, the evaluation of differences in treatment decisions was not the study aim.

### 3.1. Subgroup Analysis

Patients were divided into two subgroupsthose who completed the study at visit month 36 (*n* = 27, “36 months complete”) and those who finished the observation before month 36 (*n* = 6, “36 months incomplete”) because their injection interval was extended to 16 weeks twice. At the baseline, BCVA, age, and CRT showed no statistically significant difference between both groups (Table 1). In group “36 months complete”, there was no statistically significant increase in BCVA from the baseline (70.8 ± 12.2) to month 36 (74.4 ± 13.7; *p*=0.165). However, CRT decreased significantly from 480 ± 156.2 *μ*m at baseline to 314.7 ± 97.3 *μ*m at month 36 (*p* < 0.001). In the group “36 months incomplete”, BCVA increased significantly by 15.2 ± 9.9 letters from the baseline (68.7 ± 11.5) to the final visit (83.8 ± 4.6, *p*=0.014). The CRT in this group decreased accordingly (*p* < 0.001) from 399.8 ± 22.4 *μ*m at the baseline to 298.8 ± 29.1 *μ*m at the final visit. In terms of BCVA and CRT, no difference was observed between both groups, neither at the baseline nor at the final visit (see Table 1).

## 4. Discussion

This study evaluated the morphologic and functional outcomes of TER with aflibercept in patients with DME in a network of university clinics and outpatient practitioners. The main outcome parameter, BCVA, from the baseline to the final visit, increased significantly by 5.8 letters (±13.4, *p*=0.019), with the highest gain of letters from the baseline to month 6 with 6.3 letters (±10.0, *p*=0.001). Accordingly, the CRT decreased by 154.1 (±151.3 *μ*m, *p* < 0.001). As these results are comparable to other TER trials, this individual disease management program proved to be efficient in a healthcare setting, providing reimbursement for an intravitreal anti-VEGF treatment only in a clinical setting [5–7].

To further evaluate the characteristic differences according to the individual retreatment intervals, in a post hoc analysis, all patients could be divided mainly into 2 subgroups. In subgroup 1 (*n* = 6), following the loading dose, the retreatment interval could be extended twice to 4 months. According to the protocol, these patients finished their study participation before month 36. In this group, BCVA increased significantly by 15 letters (±9.9, *p*=0.014) from the baseline to the individual final visit. In subgroup 2 (*n* = 27), all patients received frequent retreatments throughout the study period of 36 months. In this group, BCVA increased by 4 letters (*p*=0.165), showing no statistical significance from the baseline to month 36. Those results indicate a worse visual outcome in patients with a persistent need for retreatment for DME. This is according to the findings of a study by Bressler et al. showing the persistence of DME throughout a treatment period of 3 years in some patients, correlating with a worse visual outcome [8]. Any comparison to other studies based on TER in clinical settings is confined by different inclusion criteria. In our study, representing a real-world setting, patients were included with clinically significant DME without prior treatment in the last three months.

Frequently cited phase 3 studies, such as VIVID and VISTA, included DME patients if BCVA was between 73 and 24 letters [3]. Apart from that, in these studies, a fixed dose regimen was used with fixed retreatment intervals. Another trial by Pak and co-authors based on TER with aflibercept revealed a significant and even higher increase in BCVA of 8.9 letters by month 12 compared to our study [5]. This could be due to an adjustment of the retreatment interval by 2 weeks, as opposed to 4 weeks in our study. All in all, in this optimized phase 3 study setting, the mean BCVA increased by 5.8 throughout the 3 years. Thus, the results were comparable compared to the VIBIM, although our observation period was longer (one year in VIBIM study).

In our study, we chose a maximum treatment interval of 16 weeks due to several reasonsFirst, the burden of disease is reduced by lowering the number of clinical visits and injections. Second, the vitreous half-life of aflibercept is longer than that of ranibizumab, which should reduce the number of injections [9]. As presented in the ALTAIR study, the interval of aflibercept injection can be extended to more than 12 weeks in 57–60% of the patients suffering from age related macular degeneration [10]. In eyes with DME that received bevacizumab and switched to aflibercept as a second-line therapy the treatment interval was extended to a maximum of 10 weeks, as shown in the TADI study [11]. Those patients improved visual acuity in the one year follow up.

The mean injection interval was 6.2 weeks over the observation period of 3 years, ranging from 4 to 12 weeks. The mean interval between injections significantly raised from 5 (±1.5) weeks in the first year to 8 (±4.4) weeks in the third year (*p* < 0.001). The mean number of injections per year in our study decreased significantly from 11 (±1.5) injections in the first to 7.9 (±3.3) in the third year (*p* < 0.001).

Apart from functional and morphological outcomes, this study aimed also to evaluate the efficacy of a network consisting of a university clinic and ophthalmologic practitioners. In Austria, the intravitreal anti-VEGF treatment is only reimbursed in clinical settings. This leads to an overload of follow-up visits and necessary re-treatments. The idea of the network was to reduce the burden caused by the clinical examinations and additional waiting time for patients and to outsource them to partners in external ophthalmologic offices. Following their examination and advice, patients were deferred to the university clinic for the anti-VEGF treatment. Only to evaluate the efficacy of the network in the study setting, OCT examinations were repeated in the university clinic. Retreatment criteria were increasing or still decreasing DME involving the central 6 millimeters. In 86%, the decision for retreatment was identical in the ophthalmologic offices and the university clinic. In the remaining 14%, decisions differed with the clinical retina experts tending to indicate more re-treatments compared to their partners in ophthalmological offices.

Patients' adherence to the external ophthalmologist was not as high as expected within the study group. Only 17 patients visited the external ophthalmologist more than 6 times a year in the observation period. Therefore, the incongruence of the decisions between the clinic and practitioner is rather inconclusive. Not visiting the practitioner was not an exclusion criterion. In the area where the study was conducted, the distance to the treating clinic could be rather long in individual cases, causing an additional treatment burden. It has been shown previously that the distance of the external ophthalmologist to the patient's residence has an influence on the patient's adherence to the treatment [12]. Nevertheless, it can be concluded that the treating institution, whether a centralized university clinic or the decentral practitioner, should be responsible for the whole diagnostic and therapeutic process to avoid differences in the treatment approach.

As shown in a retrospective analysis in which 28,658 eyes with DME were screened, the gain of VA in the first year correlated with the number of injections [13]. This implicates that the adherence is crucial for maintaining visual acuity in patients with DME. Research shows that patient's adherence to the anti-VEGF therapy is associated with better visual outcome in eyes with DME and nAMD [14]. Apart from that, it is known that nAMD patients show a greater compliance to the therapy than patients with DME [15]. In the VIOLET study, it was shown that in patients with DME, both TER and PRN regimens achieve similar functional results. However, fewer injections were administered in the TER group, making higher patient adherence more likely [16].

There are several limitations to this studyMorphologic findings in OCT scans were not described in detail by the ophthalmologic practitioners. It had to be determined if the criteria for a reduction or elongation of the retreatment interval were met. This could have helped to avoid inconsistency between the decisions of ophthalmic practitioners and clinical ophthalmologists. Furthermore, extending and reducing the retreatment interval by 2 instead of 4 weeks as it was conducted in this study could have improved the treatment outcomes. Nevertheless, previous studies have shown that a 4-week interval extension is a reasonable strategy to treat patients with diabetic macular edema [17, 18]. The study was performed as an uncontrolled noncomparative study. Therefore, it cannot be compared with other administration regimens such as monthly injections or pro re nata use. Also, the sample size was relatively small, for this reason, the significance of the therapeutic effect is reduced.

## 5. Conclusion

Treat and extend regimen is valuable and widely used for treating patients with diabetic macular edema, since the results are comparable with fixed dosing intervals. Patient's treatment burden can be lowered by extending the intervals, and the healthcare system can be relieved by decreasing the frequency of treatment and controls.

Adherence to the same treating institute is important to avoid differences in therapeutic decisions and may increase patient's compliance.

## Figures and Tables

**Table 1:** Age, sex, and baseline-values for BCDVA and CRT for the whole study population and for the subgroups who had their final visit at month 36 or before the month 36. The *p* value describes the difference of the visual acuity and the central retinal thickness at the baseline and at the final visit between the two groups.

	Total (*n* = 33)	Baseline value of group finishing at visit month 36 *n* = 27	Baseline value of group finishing before visit month 36 *n* = 27	*p* value
Age, years	63.4 ± 11.7	63.0 ± 10.4	64.8 ± 17.4	0.366
Male : female	11 : 22 (33/66)	9 : 18 (33/66)	2 : 4 (33/66)	1
BCDVA at baseline	70.4 ± 11.9	70.8 ± 12.2	68.7 ± 11.5	0.471
BCDVA at final visit	76.2 ± 13.0	74.4 ± 13.7	83.8 ± 4.6	0.072
CRT (OCT) at baseline	465.8 ± 145.9	480.5 ± 156.2	399.8 ± 54.5	0.82
CRT (OCT) at final visit	311.8 ± 88.6	314.7 ± 97.3	298.8 ± 29.1	0.633

## Data Availability

The data used to support the findings of this study are available from the corresponding author upon request.
